# Epidemiological profile of breast cancer in a reference center in the north region of Brazil

**DOI:** 10.61622/rbgo/2025rbgo27

**Published:** 2025-05-04

**Authors:** Daniele Carvalhais França, Agnaldo Lopes da Silva, Anisse Marques Chami, Leticia da Conceição Braga

**Affiliations:** 1 Universidade Estadual Paulista Botucatu SP Brazil Universidade Estadual Paulista, Botucatu, SP, Brazil.; 2 Hospital de Amor da Amazônia Porto Velho RO Brazil Hospital de Amor da Amazônia - Fundação Pio XII, Porto Velho, RO, Brazil.; 3 Universidade Federal de Minas Gerais Belo Horizonte MG Brazil Universidade Federal de Minas Gerais, Belo Horizonte, MG, Brazil.; 4 Rede Mater Dei de Saúde Belo Horizonte MG Brazil Rede Mater Dei de Saúde, Belo Horizonte, MG, Brazil.; 5 Instituto Mário Penna Laboratory of Translational Research in Oncology Belo Horizonte MG Brazil Laboratory of Translational Research in Oncology, Instituto Mário Penna, Belo Horizonte, MG, Brazil.

**Keywords:** Breast neoplasm, Carcinoma, Epidemiology, Amazon, North region, Brazil

## Abstract

**Objective:**

To describe the epidemiological data of women with breast cancer at a referral center in oncology in the northern region of Brazil.

**Methods:**

This is a retrospective cohort study. The study population consists of patients who were diagnosed with *in situ* or invasive BC (invasive carcinoma of no special type (ICNST) and invasive lobular carcinoma (ILC)) at the *Hospital de Amor da Amazônia*, in Porto Velho – Rondônia, between January 2012 and December 2021. The sampling plan adopted was of the convenience type. All patients who received the anatomopathological diagnosis of *in situ* or invasive BC at the *Hospital de Amor da Amazônia* from 2012 to 2021 and came from the North region were included. Exclusion criteria were non-origin from the North region and absence of diagnosis established by anatomopathological examination of breast cancer. Analysis of the database and medical records of the *Hospital de Amor da Amazônia* was carried out to collect information.

**Results:**

420 patients were included, 99.5% female, with complete elementary school (32,6%) and brown skin (68,1%). The mean age at diagnosis was 49 years. Forty-five percent were born in the northern region and 55% in other regions of Brazil. Eighty percent of tumors were invasive ductal carcinoma; 32.7% were luminal A-like, 25.1% luminal B-like, 19.4% HER2 enriched and 12.8% triple negative. When patients were subdivided by age ≤40 years and > 40 years, there was a statistically significant difference in the association with staging (p=0.000), histological type (p= 0.035), immunohistochemistry subtype (p=0.000), neoadjuvant chemotherapy (p=.000) and genetic counseling (p=0.001). The median survival was 7.99 years. The 5-year overall survival was 81%. The higher the stage, the lower the survival rate. Twenty-four distinct variants were described in patients undergoing genetic testing, 16 of uncertain significance and 8 pathogenic. Three new variants were described: *ATM* (c.8726G>C), *BRCA2* (c.2232A>C) and *ERCC5* (c.2164G>Ap).

**Conclusion:**

In this study, the age at diagnosis of breast cancer was lower, the tumor subtype was more aggressive, and patients were admitted in more advanced stages. Overall survival is lower compared to national and international data. Despite the small number of patients referred to genetic testing, it is important to search for germline mutations to improve patients’ diagnosis and treatment.

## Introduction

Among malignant neoplasms, breast cancer (BC) has become the most diagnosed in women, excluding nonmelanoma skin cancers (NMSC), and shows a growing trend, especially in developing countries. It is estimated that in 2020, BC was responsible for 16% of cancer deaths in women, and by 2040, this rate will increase by more than 50%.^(1)^ In Brazil, the numbers follow the global statistics, keeping BC ahead of other neoplasms, except NMSC, in all regions. According to the National Cancer Institute (INCA), the estimate for the three-year period from 2023 to 2025 indicates that there will be 704,000 new cancer cases per year.^(2)^

The Northern region of Brazil is the largest in territorial extension but has the second-smallest population, according to the Brazilian Institute of Geography and Statistics (IBGE).^(3,4)^ Until 2020, it was the only region in the country where rates of breast and cervical cancer were equivalent.^(5,6)^ However, the incidence of BC exceeded that of the uterine cervix from that date on, remaining in first place in the estimate for the next three-year period from 2023 to 2025, reaching 73,000 new cases (66.54/100,000).^(2)^ Despite a lower estimated incidence of BC than in other Brazilian regions, it is known that the proportion of advanced disease (stages III and IV) before the beginning of treatment is higher in the North Region (50.1%).^(7)^

Considering the impacts of BC on mortality, morbidity and in the socioeconomic context, studies regarding this pathology are essential. Due to the lack of data in the literature on BC in the North of Brazil, further studies are necessary, starting with the epidemiological profile description, the characteristics of the tumors and the behavior of the disease in this population. Thus, our objective is to describe the epidemiological data regarding women diagnosed with BC treated at a reference center in oncology in the North region of Brazil from January 2012 to December 2021.

## Methods

This is a retrospective cohort study. The study population consists of patients who were diagnosed with *in situ* or invasive BC (invasive carcinoma of no special type (ICNST) and invasive lobular carcinoma (ILC)) at the *Hospital de Amor da Amazônia*, in Porto Velho – Rondônia, between January 2012 and December 2021. The sampling plan adopted was of the convenience type. All patients who received the anatomopathological diagnosis of *in situ* or invasive BC at the *Hospital de Amor da Amazônia* from 2012 to 2021 and came from the North region were included. Exclusion criteria were non-origin from the North region and absence of diagnosis established by anatomopathological examination of breast cancer. Analysis of the database and medical records of the *Hospital de Amor da Amazônia* was carried out to collect information.

The method used for patients undergoing genetic testing was the NGS panel performed at the same institution (*Hospital de Amor de Barretos – Fundação Pio XII*), except for four tests performed at another institution. The data collected in the previously defined and standardized form were recorded on the REDCap 13.1.13 platform.^(8)^ This software complies with what is proposed by the General Law for the Protection of Personal Data (LGPD or LGPDP), Law No. 13.709/2018.^(9)^ In addition to the sociodemographic and histopathological characteristics, a comparative analysis was performed between variables and age (less than or equal to 40 years old and greater than 40 years old). The sample was characterized through frequency tables for the study variables. The chi-square or Fisher's exact tests were used to compare the groups. For analysis of patient survival, the Kaplan-Meier methodology was used, and the comparisons were done by the Logrank test. The overall survival time was from the date of diagnosis to the date of death or the date of 12/31/2021. The comparisons were based on staging, age, color/race, and immunohistochemistry subtype. Data were analyzed using SPSS version 27 software, adopting a significance level of 5% (0.05).

The present study followed ethical regulation in accordance with CNS resolution 466/2012 and was approved by the Pio XII Foundation Research Ethics Committee (Certificado de Apresentação de Apreciação Ética: 53163121.1.0000.5437, Report number: 5.174.939).

## Results

Five hundred records were selected between January 2012 and December 2021; 420 were included in the study. Seventy-nine patients were eliminated after the exclusion criteria. As shown in table 1, most patients are female (99.5%); they have some level of education, and the majority have completed elementary school (33.3%). 68% self-declared brown. The mean age at diagnosis was 49 years.; 46.6% were pre-menopause and 52.1% were post-menopause. Regarding origin, 45% were born in the North region, and 55% were born in other Brazilian regions. Between the North region population, 87.8% of the patients are from the State of Rondônia.

**Table 1:** Sociodemographic characteristics of patients with breast cancer treated

Sociodemographic characteristics		n(%)
Gender	Female	415(99,5)
	Male	2(0,5)
Education	Undefined	19(4,6)
	Iliterate	15(3,6)
	Primary school	137(33,3)
	Secondary school	93(22,6)
	University degree	80(19,4)
	Postgraduate	19(4,6)
	Uninformed	49(11,9)
Reprodutive status	Premenopause	169(46,6)
	Postmenopause	189(52,1)
	Uninformed	5(1,4)
	Not applicable	1(0,3)
Color/race	Undefined	26(6,3)
	White	74(18)
	Black	17(4,1)
	Asian	3(0,7)
	Brown	282(68,8)
	Indigenous	0(0)
	Other	2(0,5)
	Uninformed	6(1,5)
States of origin	Rondônia	366(87,8)
	Acre	32(7,7)
	Amazonas	9(2,2)
	Amapá	6(1,4)
	Pará	3(0,7)
	Tocantins	1(0,2)
	Roraima	0(0)
Place of birth	Noth	144(44,8)
	Northwest	29(9)
	South	58 (18)
	Southeast	56(17,4)
	Center-west	34 (10,6)
	**Average**	**Standard deviation**
Age at diagnosis (Years)	49	12,32

Table 2 presents the histopathological and treatment characteristics of the patients. The main histology identified is ICNST (80.9%), following 8.4% of the tumors corresponding to ductal carcinoma *in situ* (DCIS), 5.5% to special types, and 5.3% to invasive lobular carcinomas (ILC). The largest number of patients was stage II (32.5%) and III (31.7%). Regarding immunohistochemistry subtypes, 32.7% were luminal A-like, 25.1% luminal B-like, 19.4% HER2 overexpressed, and 12.8% triple negative. In the group described as not applicable, DCIS was classified. Of the patients who received chemotherapy (76.8%), 39.6% were in the neoadjuvant setting and 37.2% in the adjuvant setting; 81.5% of the patients received radiotherapy; 91.2% underwent surgery; 71.8% received hormone therapy, and 21.2% received targeted therapy for HER2-enriched tumors.

**Table 2:** Histopathological characteristics and treatment of patients with breast cancer

Clinical-pathological chacteristics		n(%)
Histology	IDC	338(80.9)
	ILC	22(5.3)
	DCIS	35(8,4)
	Special subtypes	23(5,5)
Staging	0	38(10,1)
	I	73(19,3)
	II	123(32,5)
	III	120(31,7)
	IV	24(6,3)
Immunohistochemistry subtype	Luminal A-like	133(32,7)
	Luminal B-like	102(25,1)
	Her 2 enriched	79(19,4)
	Triple negative/basal like	52(12,8)
	Not applicable	37(9,1)
	Uninformed	4(1,0)
Neoadjuvant chemotherapy	No	184(46,1)
	Yes	158(39,6)
	Not applicable	55(13,8)
	Uninformed	2(0,5)
Adjuvant chemotherapy	No	159(45,2)
	Yes	131(37,2)
	Not applicable	58(16,5)
	Uninformed	4(1,1)
Radiotherapy	No	45(12,8)
	Yes	286(81,5)
	Not applicable	14(4)
	Uninformed	6(1,7)
Surgery	No	27(6,6)
	Yes	373(91,2)
	Not applicable	7(1,7)
	Uninformed	2(0,5)
Endocrine therapy	No	98(27,5)
	Yes	256(71,8)
	Uninformed	3(0,8)
Target therapy	Yes	82(21,2)
	No	304(78,6)

The patients were subdivided into ages less than or equal to 40 years and greater than 40 years and correlated with the patients’ data, as shown above in table 3. There was a statistically significant difference in the association with staging (p=0.000), histological type (p= 0.035), Immunohistochemistry subtype (p=0.000), neoadjuvant chemotherapy (p= 0.005) and genetic counseling (p=0.001).

**Table 3:** Correlation between age and socioeconomic, histopathological and treatment variables of patients with breast cancer treated

		Age		p-value
		≤40 years	>40 years	
		n(%)	n(%)	
Color/race	Undefined	6(6,3)	20(6,4)	0.542
	White	15(15,6)	58(18,6)	
	Black	1(1)	15(4,8)	
	Asian	1(1)	2(0,6)	
	Brown	71(74)	210(67,5)	
	Indigenous	0(0)	0(0)	
	Other	0(0)	2(0,6)	
	Uninformed	2(2,1)	4(1,3)	
Education	Undefined	6(6,2)	13(4,2)	. ^b^
	Iliterate	0(0)	15(4,8)	
	Primary school	20(20,6)	116(37,1)	
	Secondary school	29(29,9)	63(20,2)	
	University degree	25(25,8)	54(17,3)	
	Postgraduate	5(5,1)	14(4,5)	
	Uninformed	12(12,4)	37(11,9)	
Staging	0	4(4,4)	33(11,6)	0,000
	I	7(7,7)	66(23,2)	
	II	30(33)	92(32,4)	
	III	43(47,3)	76(26,8)	
	IV	7(7,7)	17(6)	
Immunohistochemistry subtype	Luminal A-like	18(19,1)	114(36,8)	0,000
	Luminal B-like	25(26,6)	77(24,8)	
	Her 2 enriched	20(21,3)	58(18,7)	
	Triple negative/basal like	26(27,7)	26(8,4)	
	Not applicable	4(4,3)	32(10,3)	
	Uninformed	1(1,1)	3(1)	
Neoadjuvant chemotherapy	No	31(33,3)	152(50,2)	0,005
	Yes	51(54,8)	106(35)	
	Not applicable	11(11,8)	43(14,2)	
	Uninformed	0(0)	2(0,7)	
Genetic couseling	No	56(61,5)	272(95,8)	0,001
	Yes	35(38,5)	12(4,2)	

**Figure 1:**
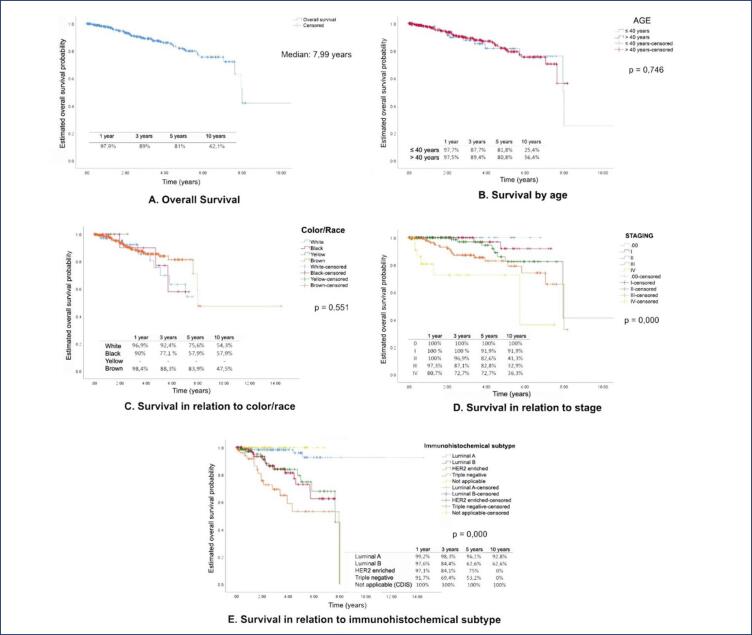
Analysis of overall survival and relationship with variables of patients with breast cancer treated

The overall survival and their relationship with clinical data of the evaluated patients was performed, as shown in figure 1. The median was 7.99 years. Overall survival at 1 year was 97.9%, 3 years 89%, 5 years 81% and 10 years 42.1%. Regarding age less than or equal to 40 years and greater than 40 years, there was no statistically significant difference, as well as when comparing survival between color/race groups and time between diagnosis and initiation of treatment. Survival in relation to staging showed a statistically significant difference, with lower overall survival the higher the stage. Stage 0 had 100% survival, while stages I, II, and III had 91.9%, 41.3%, and 32.9% in 5 years, respectively. The 3-year survival of stage IV was 36.3%.

Likewise, survival in relation to Immunohistochemistry subtypes was also significant. At 5 years, survival was 96.1% for luminal A-like, 62.6% for luminal B-like, 75% for overexpressed HER2, 53.2% for triple-negative/basal-like, and 100% for DCIS. Table 4 shows the main mutations found in the 43 patients who underwent genetic counseling and underwent some type of genetic test for germline mutation research. The method used was the NGS panel performed at the same institution (*Hospital de Amor de Barretos – Fundação Pio XII*), except for four tests performed at another institution (two negative tests, one test showing a variant of uncertain significance (VUS) in the *MSH2* gene and the other showing 4 different VUS in the same patient (*TSC2*, *ERCC5* and *FANCL* genes). Of the 43 genetic tests performed, 24 distinct variants were reported. In three patients, more than one variant was found. One with a pathogenic variant in *BRCA1* (c.181T>G) and a VUS in *BRCA2* (c.6940A>G); one with four VUS (*TSC2* (c.6940A>G), *TSC2* (c.4216G>A), *ERCC5* (c.2164G>Ap) and *FANCL* (c.1096_1099dup)) and another with a pathogenic variant in CHEK2 (exon deletion 9) and VUS in *BRCA1* (Duplication of exons 1 and 2). Those mutations involving entire exons were confirmed by MLPA (Multiplex Ligation-dependent Probe Amplification). Eight variants were considered pathogenic, and 16 were classified as VUS. The mutation in *ERCC5* (c.2164G>Ap) is new. The most affected genes were *BRCA1, BRCA2,* and *ATM*. The main change observed was the substitution of a single base (SNVs). Three new variants have been described: *ATM* (c.8726G>C), *BRCA2* (c.2232A>C) and *ERCC5* (c.2164G>Ap).

**Table 4:** Main mutations identified in patients who underwent genetic testing for germline

Variant subtype	Gene	Identified variant	Phenotypic effect	Genotype
Pathogenic	RAD51C*	c.404C>A	(p.Cys135Tyr)	Heterozygosity
	BRCA1*	c.181T>G	(p.Cys61Gly)	
	*BRCA1*	c.5463_5464insT	(p.His1822fs)	
	*BRCA2*	c.3975_3978dup	(p.Ala1327fs)	
	*ATM*	c.8814_8824del	(p.Met2938fs)	
	*PALB2*	c.43G>T	(p.Glu15Ter)	
	*PMS2*	c.2182_2184delinsG	(p.THr728Alafs)	
	CHEK2*** + **	Deletion of exon 9		
Variants of uncertain significance (VUS)	*ATM*	c.8726G>C	(p.Arg2909Thr)	
	*ATM*	c.4012A>G	(p.Ser1338Gly)	
	BRCA1***	Duplication of exons 1 and 2		
	*BRCA2*	c.8942A>G	(p.Glu2981Gly)	
	*BRCA2*	c.2232A>C	(p.Ser744=)	
	BRCA2*	c.6940A>G	(p.Thr2314Ala)	
	*CHEK2*	c.962A>C	(p.Glu321Ala)	
	TSC2**	c.661G>A	(p.Val221Met)	
	TSC2**	c.4216G>A	(p.Asp1406Asn)	
	ERCC5**	c.2164G>Ap	(p.Glu722Lys)	
	FANCL**	c.1096_1099dup	(p.Thr367fs)	
	*MSH2*	c.605C>T	(p.Pro202Leu)	
	*MLH1*	c.1487C>G	(p.Pro496Arg)	
	*MSH6*	c.3209G>A	(p.Gly1070Asp)	
	*PMS2*	c.1004A>G	(p.Asn335Ser)	
	*BRIP1*	c.545A>G	(p.Asn182Ser)	

*, **, *** mutations found in the same patient;

+ variant confirmed by MLPA

## Discussion

Breast cancer is the most diagnosed tumor in women, excluding non-melanoma skin cancer, and shows a growing trend, especially in developing countries. In Brazil, this reality is no different. In all Brazilian regions, breast cancer ranks first in incidence.^(2)^ Data regarding breast cancer in the North region of Brazil are quite scarce.

In this study, 420 patients diagnosed with breast cancer and from the northern region of Brazil were evaluated. Patients from all northern states were included, except for Roraima. Most came from Rondônia (87.8%), followed by Acre (7.7%) and Amazonas (2.2%). Below the national and world average the mean age at diagnosis was 49 years. Data from SEER (Surveillance, epidemiology, and end results program) shows that the average age at diagnosis in the United States is 63 years.^(10)^ In Brazil, as described by Franzoi et al.,^(11)^ the age range is between 53 and 55 years.

In line with other studies in the North region, most patients had at least the first degree and declared themselves brown.^(12–16)^ It was also observed that there was no self-declared indigenous person in the study sample. Surprisingly, in terms of place of birth, 55% of the patients were not born in the North region. Of these, 18% were born in the South, 17% in the Southeast, 11% in the Midwest and 9% in the Northeast. It was expected to find a greater indigenous and Latin American people influence however, this was not the result found in this study. These data are in line with the publication by Vieira et al.,^(17)^ who described the Brazilian ancestry and showed that the greatest influence in all regions is indeed European (0.700 ± 0.212), followed by African (0.160 ± 0.171), Amerindian (0.071 ± 0.076), and Asian (0.067 ± 0.101).

When the patients are divided by age less than or equal to 40 years and greater than 40 years, younger patients were diagnosed with more advanced staging (EC III = 47.3%), while those over 40 years old are diagnosed at an early stage. (EC II= 32.4%). These results differ from those presented in the AMAZONA III study, which also evaluated patients by age throughout Brazil, in the same way as the present study. In it, both patients under 40 years old and over 40 years old were mostly diagnosed in initial stages (EC II). Also, in comparison with this work, young patients were mostly classified as luminal B-like (15,8% x 11,4%) and older patients as luminal A-like (51,3% x 30,6%).^(11)^ Here in the northern region, the highest proportion among young patients was of triple negative tumors (27.7%), corroborating the greater aggressiveness of the lesions.

Regarding overall survival, 81% of patients were alive at 5 years, a lower percentage in relation to SEER data (90.8%).^(10)^ Fujimoto et al.,^(15)^ described an overall survival rate in women in the state of Acre of 87, 3%1 and Cruz et al.,^(18)^ in Pará, 79.4%. In the AMAZONA study, overall survival was 88.74%.^(12,14,18)^ Overall survival by staging followed the trend that the higher the stage, the shorter the survival. Patients diagnosed with Stage 0 disease had 100% survival, while stages I, II and III had 91.9%, 41.3% and 32.9%, in 5 years, respectively. The 3-year survival of stage IV was 36.3%.

When evaluating survival by molecular subtype at 5 years, there was statistical significance. In luminal A-like subtypes, survival was 96.1%, in luminal B-like, 62.6%. In the more aggressive subtypes, survival was worse. The overall survival at 5 years was 75% in HER2-enriched tumors and 53,2% in the triple negative/basal like patients. On the other hand, national data presented in AMAZONA III, this work showed that in stages I, there was no statistical significance in relation to tumor subtypes. In stages II and III, there was a statistically significant correlation, and the survival was worse. In stage II, HER2-enriched and triple negative tumors had 82.3% and 86.8% survival, respectively, while luminal A-like, 97.6%. In stage III, HER2-enriched, 64.1%, triple negative, 56.1%, and luminal A-like, 75.8%.^(12)^

In this study, genetic counseling with testing had been performed so far in 43 patients. Of the total number of tests that showed some variants, 7 pathogenic/probably pathogenic and 16 VUS. The main altered genes were: *BRCA1*, *BRCA2*, associated with high penetrance and increased risk of Hereditary Breast and Ovarian Cancer syndrome (HBOC); *ATM, PALB2, CHECK2, RAD5C, BRIP1* with moderate or low penetrance and other less common ones such as *MLH1, MSH6, MSH2, PMS2, TSC2, ERCC5, FANCL*.^(19)^ Three new variants have been described: *ATM* (c.8726G>C), *BRCA2* (c.2232A>C) and *ERCC5* (c.2164G>Ap), no variants recurrence was observed.

Similarly, Dobbin et al.^(20)^ investigated 15 polymorphisms in the *BRCA1* and *BRCA2* genes in Amazonian ameridians and compared them with findings from the global population. It was shown that three *BRCA2* gene variants associated with risk for hereditary breast cancer (rs11571769, rs144848 and rs11571707) had a higher frequency in these individuals compared to African, American, European, and Asian groups.

Other mutations prevalent in further studies in Brazil, such as BRCA1 c.3331_3334delCAAG, BRCA1 c.211A >G, BRCA2 c.2808_2811delACAA and BRCA2 c.5946ddelT, previously described, were not identified in this research.^(21,22)^ The founder mutationTP53 c.1010G > A (p.R337H), associated to Li-Fraumeni Syndrome which has a high frequency in Southern and Southeastern regions, was neither identified.^(23)^

Despite the small number of patients referred for genetic counseling, it is important to research and evaluate germline mutations in this population. This work associated with the study of family history and ancestry can contribute to the identification of specific pathogenic variants and the targeting of specific care for these groups.

Regarding ancestry, Vieira et al.^(17)^ evaluated differences in this aspect, in addition to self-reported ethnicity and molecular subtypes of breast cancer in the five Brazilian regions. The main ancestry across the country was European, followed by African. The north region followed the general pattern, with 61% of European ancestry and 21% of African ancestry – a higher percentage than in other regions of the country. This data follows this work in which a large portion of the natural population from regions other than the north was identified. African ancestry was associated with more triple negative and HER 2 positive tumors.^(16)^ These subtypes are associated with a higher frequency in young patients, which was also possible to identify in this study.^(17)^

Some weaknesses of this work should be refined for future publications. The database can contain a larger N of patients, aiming at a large registry in the North region. Thus, more patients from all states can be added, making the data more homogeneous.

Unexpectedly, the indigenous population was not represented in this study. This may probably be due to difficult access to communities and more restricted assistance policies that reduce the presence of this population in reference units. Although the center of this study has mobile devices that offer screening tests, there is no coverage data, as well as data on tertiary and quaternary care in the indigenous population. Like this one, other studies also had a reduced sample of this population (Suleiman et al.^(13)^ n=13; Rocha et al.^(16)^ n=1; other studies did not specify an indigenous population).

The availability of a geneticist at the service was intermittent over the years and depended on the service's headquarters in Barretos. Recently, with the hiring of an oncogenetics specialist, more patients will undergo genetic counseling and testing, generating better knowledge of germline mutations in the region.

## Conclusion

Considering the results of the work, it was possible to conclude that in the North region the age at diagnosis of breast cancer is lower than in general and tumors of a more aggressive subtype (triple negative/basal like) are more prevalent. In addition, patients are admitted at more advanced stages and survival is lower compared to national and international data. With aim to mitigate this situation, efforts to diagnose the disease early and facilitate navigation processes are important for patients to be able to treat this curable disease. In addition, family history assessment and genetic counseling should also be included more frequently when initiating treatment. In conclusion, in addition to the results shown in this study, more information can be taken from this large database, enabling the improvement of care for patients with breast cancer in the North region of Brazil.
